# Novel robust control of a 7-DOF exoskeleton robot

**DOI:** 10.1371/journal.pone.0203440

**Published:** 2018-09-07

**Authors:** Mehran Rahmani, Mohammad Habibur Rahman

**Affiliations:** Mechanical Engineering Department, University of Wisconsin-Milwaukee, Milwaukee, WI, United States of America; Shandong University of Science and Technology, CHINA

## Abstract

This paper proposes a novel robust control method for the control of a 7-DOF exoskeleton robot. The external disturbances and unknown dynamics in the form of friction forces, different upper-limb’s mass, backlash, and input saturation make robot unstable, which prevents the robot from correctly following the defined path. A new fractional sliding mode controller (NFSMC) is designed, which is robust against unknown dynamic and external disturbances. Fractional PID controller (FPID) has high trajectory tracking, but it is not robust against external disturbances. Therefore, by combining NFSMC and FPID controllers, a new compound fractional PID sliding mode controller (NCFPIDSMC) is proposed, which benefits high trajectory tracking of FPID and robustness of NFSMC. The stability of the proposed control method is verified by Lyapunov theory. A random noise is applied in order to confirm the robustness of the proposed control method.

## Introduction

Strokes are the main leading cause of death in the world and can be considered the leading cause of acquired disability in adults. The survivors from strokes commonly do not have the means to perform daily activities such as dressing, eating, and bathing by themselves because of their disability [1,2]. In recent years different researches have been done for developing of robotics devices for rehabilitation, specially for the neurorehabiliation of post-stroke patients [3]. Because of complex arm movements of human arm in nature, designing a convenient control system is the most important task. Several control methods were obtained by using modern control schemes such as robust control method [4,5], optimal control approach [6], conventional PID control law [7], and other types of controllers [8,9]. An appropriate control method for high tracking performance is required for the exoskeleton robot. Because human arm movements are completely nonlinear in nature, a conventional linear control methods are not convenient for that kind of system. Sliding mode control method is a robust against external disturbances. Because exoskeleton robots have highly complex structure, unmodel dynamic creates some uncertainties, which is not convenient for system. In addition, friction forces, different upper-limb’s mass, backlash, and input saturation make robot unstable. Therefore, many researchers have been used sliding mode control in order to control of an exoskeleton robot because of its robustness against external disturbances and unmodel dynamics. Kang et al. proposed an adaptive method for a class of 5 DOF exoskeleton robot. The proposed adaptive controller is designed to be robust against external disturbances and uncertainties [10]. Brahmi et al. proposed a novel adaptive visual tracking control method based on sliding mode control in cartesian space implemented to an exoskeleton robot [11]. Also, a new adaptive control based on nonlinear sliding mode control was proposed for control of exoskeleton robot. The proposed control law does not require well-defined dynamic and kinematic models of the system robot [12]. In following, they applied backstepping method with time delay approximation to be robust against external disturbances. The effectiveness of the proposed control method was verified experimentally [13]. Madani et al. proposed a fast terminal sliding mode technique for articulated systems represented by exoskeleton to perform flexion/extension movements. The proposed robust controller has been implemented experimentally to derive a 3 DOF upper limb exoskeleton [14]. Riani et al. proposed an adaptive integral sliding mode control law applied to an upper limb exoskeleton. The proposed control method demonstrated high tracking performance when assisting wearers with shoulder, elbow and wrist joint movements [15]. Brahmi et al. proposed a new adaptive super-twisting control for an exoskeleton robot with dynamic uncertainties. An exoskeleton robot that is compatible with the human arm configuration and is able to obtain different rehabilitation movements and assistive tasks is proposed [16]. FSMC has better performance in comparison with conventional sliding mode control in terms of robustness and stability. Because FSMC is robust against external disturbances, it has been used in different structures. It can be taken into consideration that design of fractional sliding mode surface is highly important in FSMC. Guo and Ma proposed a novel fractional global sliding mode control method. The proposed control method ensured global stabilaztion of the system and reduction of the chattering phenomenon during the control processes [17]. Munoz-Vazquez et al. proposed a novel fractional-order controller based on sliding mode. The proposed controller includes two advantages such as robustness against external disturbances and a principle of dynamic memory resetting of the differintegral operator [18]. Zhang et al. suggested a new fractional- order sliding mode control method based on a linear-quadratic regulator (LQR) for a class of uncertain systems. In order to linearize the nonlinear system and decouple tracking error dynamics, input/output feedback linearization is used. Then, by designing LQR, the tracking error dynamics converges to the equilibrium point. The simulation results demonstrated that the proposed control scheme shows excellent performance and robustness with system uncertainties [19].

The main drawbacks of FSMC is creating chattering phenomenon, which is not appropriate for a system. Therefore, by regarding this issue, many hybrid control method is proposed in order to reduce chattering phenomenon. Razzaghian and Moghaddam proposed a fuzzy sliding mode controller for position tracking of a 5 DOF upper-limb robot. The simulation results confirmed that applied fuzzy control eliminate chattering phenomenon caused by sliding mode controller [20]. Babaiasl et al. proposed a sliding mode control whose, parameters were tuned with genetic algorithms [21]. Wu et al. developed a modified sliding mode control method with PID sliding surface. The chattering phenomenon of PID sliding mode reduced by using fuzzy control [22]. Mushage et al. proposed an adaptive nonlinear control method, which uses a new reaching law-based sliding mode control technique. This method incorporates a high-gain state observer with dynamic high-gain matrix and a fuzzy neural network for state observer and nonlinear dynamic estimation, repectively. Simulation results illustrated that the proposed control method includes benefits such as: faster response, fewer oscillation during transient phase, good tracking accuracy and chattering-free control torque with lower amplitudes [23].

In this paper, NCFPIDSMC control method for a 7-DOF exoskeleton robot is proposed. The main motivations in the paper are highlighted as follows:

1. A new fractional sliding surface is designed to enhance the robustness of the proposed control system.
2. A novel compound control method is applied, which benefits from the robustness of NFSMC and from high tracking performance of FPID.

Consequently, a new compound control method is proposed, which benefits both high trajectory tracking of FPID and robustness of NFSMC for control of an upper-limb exoskeleton robot.

The rest of this paper is arranged as follows. In Section 2, model dynamics describtion of the 7-DOF exoskeleton robot is presented. In Section3, the NFSMC is included. In Section 4, NCFPIDSMC is included. In section 5, the implementaion of fractional-order operator is described. Section 6: presents simulation results. Finally, Section 7 provides the conclusion and contributions of the work.

## Characterization of system rehabilitation

### Exoskeleton robot development

**Fig 1:**
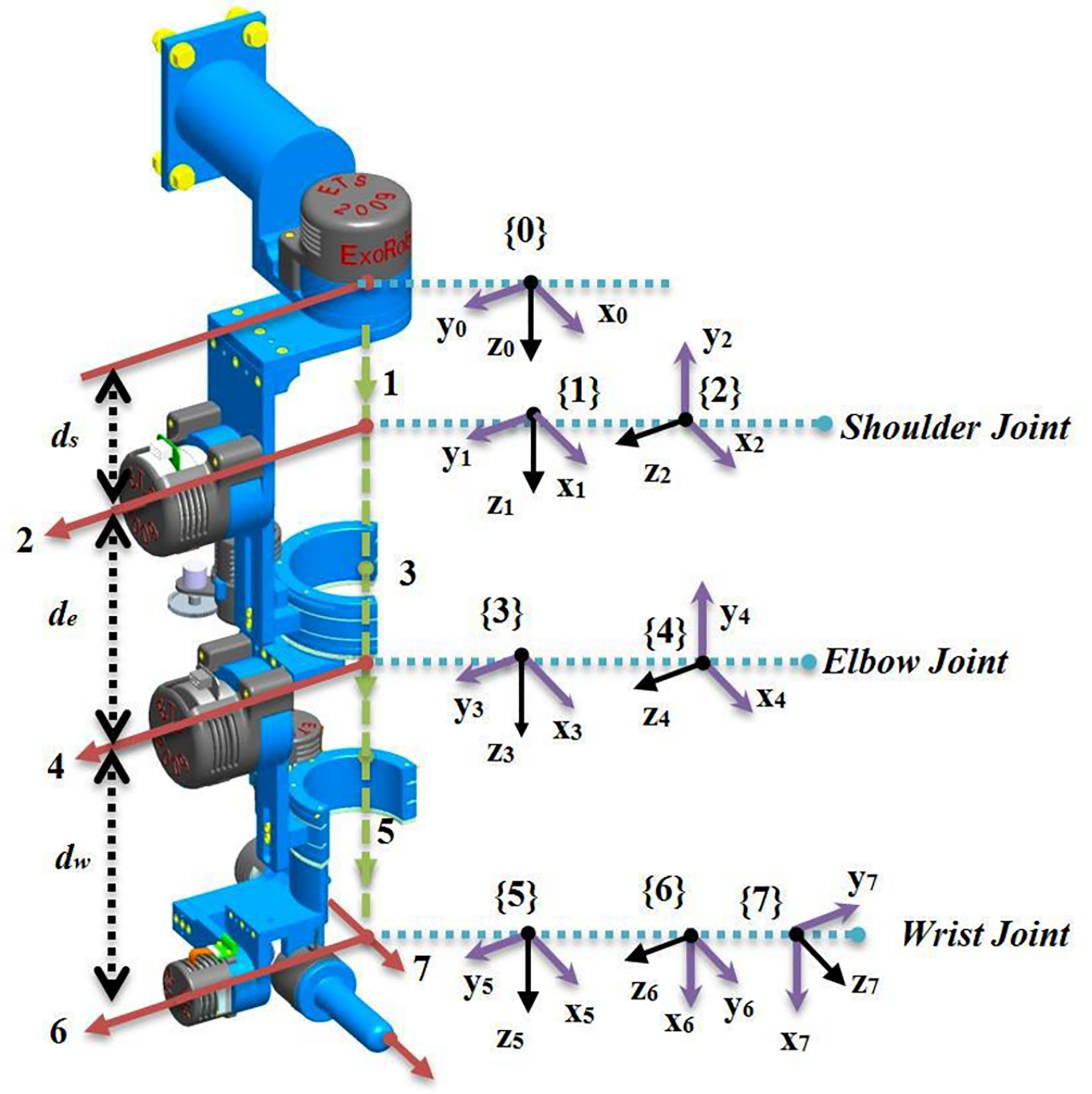
Reference frames of ETS-MARSE.

The proposed control method applied on exoskeleton robot ETS- Motion Assistive Robotic-exoskeleton for Superior Extremity (ETS- MARSE), which is a redundant robot including Seven DOFs (Fig 1). Patients whose upper limb was injured, the ETS- MARSE robot was developed to help them in physical therapy and assisted motion. By inspiring from anatomy of human upper limb, the idea of exoskeleton robot was generated. The shoulder part includes three joints; the elbow part comprises one joint, and the wrist part includes three joints. According to Table 1, each part performs different upper-limb motions. The most important advantages of ETS-MARSE can be enumerated as low weight, a convenient power/weight ratio, capable of compensating for gravity, and easily fitted or removed. Because of the length of the ETS-MARSE adjustable links, the robot can be utilized in variety aspects. The proposed robot can perform active assistive motion (electromyography, respond to force, and/or be compliant with the subject to accompany and assist humans in the intended motion) and passive (completely support and perform the motion on the subjects upper limb). The characteristics of ETS-MARSE robot are completely summarized in [24–27].

**Table 1:** Workspace ETS-MARSE.

Joints	Motion	Workspace
1	Shoulder joint horizontal flexion/extension	0°/140°
2	Shoulder joint vertical flexion/extension	140°/0°
3	Shoulder joint internal/external rotation	-85°/75°
4	Elbow joint flexion/extension	120°/0°
5	Forearm joint pronation/supination	-85°/85°
6	Wrist joint ulnar/radial deviation	-30°/20°
7	Wrist joint flexion/extension	-50°/60°

### Kinematics of ETS-MARSE robot

The Jacobian matrix is defined as the transformation from joint to Cartesian space. By using the pseudo-inverse of the Jacobian, the inverse kinematics can be obtained because of the redundant nature of ETS-MARSE robot, which can be defined as follows:

$$\dot{\theta }=\left(J^{T}{\left(JJ^{T}\right)}^{-1}\right){\dot{x}}_{d}$$ (1)

Where ${\dot{x}}_{d}$ is the desired Cartesian velocity, $\dot{\theta }$ is calculated joint velocity and *J* is the Jacobian matrix of the robot. The modified Denavit-Hartenberg (DH) parameters are given in Table 2 [28]. These parameters are used to obtain the homogeneous transformation matrices, which are obtained from frames reference illustrated in Fig 1. The workspace of the designed robot is given in Table 1. The more explanation of ETS-MARSE robot design and further detailed information of the parameters can be found in [8].

**Table 2:** Modified Denavit- Hartenberg parameters.

*Joint(i)*	*a*_*i-1*_	*a*_*i-1*_	*d*_*i*_	*Ɵ*_*i*_
1	0	0	*d*_*s*_	*Ɵ*_*1*_
2	-π/2	0	0	*Ɵ*_*2*_
3	π/2	0	*d*_*e*_	*Ɵ*_*3*_
4	-π/2	0	0	*Ɵ*_*4*_
5	π/2	0	*d*_*w*_	*Ɵ*_*5*_
6	-π/2	0	0	*Ɵ*_*6*_-π/2
7	-π/2	0	0	*Ɵ*_*7*_

### Dynamic modeling of the ETS-MARSE

The dynamic equation of the ETS-MARSE in joint space is defined as follows:

$$M(\theta )\ddot{\theta }+C(\theta ,\dot{\theta })\dot{\theta }+G(\theta )+F(\theta ,\dot{\theta })+E(t)=\tau$$ (2)

Where *M* (*θ*) ∈ *R*^7×7^, $C(\theta ,\dot{\theta })\in R^{7}$, and *G* (*θ*) ∈ *R*^7^ are the symmetric positive definite inertia matrix, the Coriolis and Centrifugal matrix, and the gravitational vector considering both the user’s arm and the exoskeleton arm, respectively, *θ* ∈ *R*^7^, $\dot{\theta }\in R^{7}$, and $\ddot{\theta }\in R^{7}$ are the joints position, velocity, and acceleration vectors, respectively. *τ* ∈ *R* is the torque input vector, *E*(*t*) ∈ *R*^7^ is the external disturbances vector, and $F(\theta ,\dot{\theta })\in R^{7}$ is the friction vector.

Obviously, Eq (2) can be denoted as follows:

$$\ddot{\theta }=-V\dot{\theta }-WG-XF-YE+Z\tau$$ (3)

Where $V=M^{-1}(\theta )C(\theta ,\dot{\theta })$, *W* = *X* = *Y* = *Z* = *M*^−1^(*θ*). Δ*V*, Δ*W*, Δ*X*, Δ*Y*, and Δ*Z* present some uncertainties of parameter variations. Therefore, Eq (3) can be rewritten as follows:

$$\ddot{\theta }=-(V+\Delta V)\dot{\theta }-(W+\Delta W)G-(X+\Delta X)F-(Y+\Delta Y)E+(Z+\Delta Z)\tau$$ (4)

Subscripts *l* and *u* stand for the lower and upper uncertainty values respectively. Where the uncertainties can be bounded as follows:

$$\begin{array}{l} \left|\Delta V_{l}\right|<\left|\Delta V\right|<\left|\Delta V_{u}\right|,\left|\Delta W_{l}\right|<\left|\Delta W\right|<\left|\Delta W_{u}\right|,\left|\Delta X_{l}\right|<\left|\Delta X\right|<\left|\Delta X_{u}\right|\\ ,\left|\Delta Y_{l}\right|<\left|\Delta Y\right|<\left|\Delta Y_{u}\right|,and\;\left|\Delta Z_{l}\right|<\left|\Delta Z\right|<\left|\Delta Z_{u}\right| \end{array}$$ (5)

## New fractional sliding mode control

Fractional differential equations have become a strong tool in describing the dynamics of complex systems. Fractional sliding mode controller has been widely used in recent years. It has been designed in order to tackle with modeling inaccuracies and external noises which are unavoidable in the real-world application. The ETS-MARSE will be encountered with modeling uncertainties and external disturbances. Therefore, by designing a NFSMC, a robust controller can be used for the ETS-MARSE robot. The proposed new fractional sliding mode surface can be defined as follows:

$$s(t)=\dot{e}+\alpha D^{\mu }e+\lambda \int_{0}^{t}e{\left(t\right)}^{2}sign(e(\tau ))d\tau$$ (6)

Where *λ* and *α* are positive constants, *μ* is fractional order operator, and *D = d/dt*. The fractional operator type is Geunwald-Letnikov operator. The tracking error is defined as:

$$e(t)=\theta_{d}-\theta$$ (7)

Take the derivative of sliding surface with respect to time in order to obtain equivalent as follows:

$$\dot{s}(t)=\ddot{e}+\alpha \mu D^{\mu +1}e+\lambda e{\left(t\right)}^{2}sign(e(t))$$ (8)

By substituting Eq (4) into Eq (8), the Eq (8) can be written as follows:

$$\begin{array}{l} \dot{s}(t)={\ddot{\theta }}_{d}+(V+\Delta V)\dot{\theta }+(W+\Delta W)G+(X+\Delta X)F+(Y+\Delta Y)E\\ \;-(Z+\Delta Z)\tau +\alpha \mu D^{\mu +1}e+\lambda e(t{)}^{2}sign(e(t)) \end{array}$$ (9)

By regarding *τ* = *u*(*t*), the control effort is derived as the solution of $\dot{s}(t)=0$ without consedering uncertainty (*E(t) = 0*). The control effort can be obtained as:

$$u_{eq}(t)=Z^{-1}({\ddot{\theta }}_{d}+V\dot{\theta }+WG+XF+YE+\alpha \mu D^{\mu +1}e+\lambda e{\left(t\right)}^{2}sign(e(t)))$$ (10)

When unpredictable perturbations from external disturbances or parameter variations occur, the equivalent control effort cannot guarantee the favorable performance. As a result of this, auxiliary control effort should be proposed in order to eliminate the effect of external disturbances. The Lyapunov function should be chosen in order to solve this problem as follows [29–31]:

$$L(t)=\frac{1}{2}s^{T}(t)s(t)$$ (11)

In order to guarantee that the control method is stable, a sufficient condition can be defined as follows:

$$\dot{L}(t)=s^{T}\;(t)\dot{s}(t),\;s(t)\neq 0$$ (12)

By completing the equivalent control ueq(t) given in Eq (10) through adding us(t), the reaching condition can be satisfied:

$$u(t)=u_{eq}(t)+u_{s}(t)$$ (13)

To obtain the reaching control signal *u*_*s*_*(t)*, Eq (12) is denoted as follows:

$$\dot{L}(t)=s^{T}\;(\ddot{e}+\alpha \mu D^{\mu +1}e(t)+\lambda e{\left(t\right)}^{2}sign(e(t)))$$ (14)

By using $\ddot{e}={\ddot{\theta }}_{d}-\ddot{\theta }$, Eq (14) can be written as follows:

$$\dot{L}(t)=s^{T}\;({\ddot{\theta }}_{d}-\ddot{\theta }+\alpha \mu D^{\mu +1}e(t)+\lambda e{\left(t\right)}^{2}sign(e(t)))$$ (15)

By substituting Eq (4) into Eq (15), it can be denoted as follows:

$$\begin{array}{l} \dot{L}(t)=s^{T}({\ddot{\theta }}_{d}+(V+\Delta V)\dot{\theta }+(W+\Delta W)G+(X+\Delta X)F+(Y+\Delta Y)E\\ \;-(Z+\Delta Z)u(t)+\alpha \mu D^{\mu +1}e(t)+\lambda e(t{)}^{2}sign(e(t))) \end{array}$$ (16)

By substituting Eq (13) into Eq (16), it can be shown as:

$$\begin{array}{l} \dot{L}(t)=s^{T}({\ddot{\theta }}_{d}+(V+\Delta V)\dot{\theta }+(W+\Delta W)G+(X+\Delta X)F+(Y+\Delta Y)E\\ \;-(Z+\Delta Z)u_{eq}(t)-(Z+\Delta Z)u_{s}(t)+\alpha \mu D^{\mu +1}e(t)+\lambda e(t{)}^{2}sign(e(t))) \end{array}$$ (17)

By substituting Eq (10) into Eq (17), it can be shown as follows:

$$\begin{array}{l} \dot{L}(t)=s^{T}({\ddot{\theta }}_{d}+(V+\Delta V)\dot{\theta }+(W+\Delta W)G+(X+\Delta X)F+(Y+\Delta Y)E\\ \;-(Z+\Delta Z)(Z^{-1}({\ddot{\theta }}_{d}+V\dot{\theta }+WG+XF+YE+\alpha \mu D^{\mu +1}e+\lambda e(t{)}^{2}sign(e(t))))\\ \;-(Z+\Delta Z)u_{s}(t)+\alpha \mu D^{\mu +1}e(t)+\lambda e(t{)}^{2}sign(e(t))) \end{array}$$ (18)

Eq (18) can be arranged as follows:

$$\begin{array}{l} \dot{L}(t)=s^{T}({\ddot{\theta }}_{d}+V\dot{\theta }+\Delta V\dot{\theta }+WG+\Delta WG+XF+\Delta XF+YE+\Delta YE-{\ddot{\theta }}_{d}-V\dot{\theta }\\ \;-WG-XF-YE-\alpha \mu D^{\mu +1}e(t)-\lambda e(t{)}^{2}sign(e(t))-Z^{-1}\Delta Z{\ddot{\theta }}_{d}\\ \;-Z^{-1}\Delta ZV\dot{\theta }-Z^{-1}\Delta ZWG-Z^{-1}\Delta ZXF-Z^{-1}\Delta ZYE\\ \;-Z^{-1}\Delta Z\alpha \mu D^{\mu +1}e(t)-Z^{-1}\Delta Z\lambda e(t{)}^{2}sign(e(t))\\ \;-(Z+\Delta Z)u_{s}(t)+\alpha \mu D^{\mu +1}e(t)+\lambda e(t{)}^{2}sign(e(t))) \end{array}$$ (19)

Simplifying Eq (19) results in

$$\begin{array}{l} \dot{L}(t)=s^{T}(\Delta V\dot{\theta }+\Delta WG+\Delta XF+\Delta YE-Z^{-1}\Delta Z{\ddot{\theta }}_{d}-Z^{-1}\Delta ZV\dot{\theta }\\ \;-Z^{-1}\Delta ZWG-Z^{-1}\Delta ZXF-Z^{-1}\Delta ZYE-Z^{-1}\Delta Z\alpha \mu D^{\mu +1}e(t)\\ \;-Z^{-1}\Delta Z\lambda {\left|e(t)\right|}^{2}sign(e(t))-(Z+\Delta Z)u_{s}(t))\\ \;\leq s^{T}(-\left|Z^{-1}\Delta Z\right|\left|{\ddot{\theta }}_{d}\right|-\left|Z^{-1}\Delta ZV+\Delta V\right|\left|\dot{\theta }\right|+\left|\Delta W-Z^{-1}\Delta ZW\right|\left|G\right|\\ \;+\left|\Delta X-Z^{-1}\Delta ZX\right|\left|F\right|+\left|\Delta Y-Z^{-1}\Delta ZY\right|\left|E\right|-\left|Z^{-1}\Delta Z\alpha \mu D^{\mu +1}e(t)\right|\\ \;-\left|Z^{-1}\Delta Z\lambda e{\left(t\right)}^{2}sign(e(t))\right|)-s^{T}((Z+\Delta Z)u_{s}(t)) \end{array}$$ (20)

To ensure Eq (20) is less than zero, the reaching control law should be chosen as follows:

$$\begin{array}{l} u_{s}(t)=sign(s)(Z+\Delta Z{)}^{-1}(\left|Z^{-1}\Delta Z_{l}\right|\left|{\ddot{\theta }}_{d}\right|+\left|Z^{-1}\Delta Z_{l}V+\Delta V_{l}\right|\left|\dot{\theta }\right|\\ \;+\left|\Delta W_{u}-Z^{-1}\Delta Z_{u}W\right|\left|G\right|+\left|\Delta X_{u}-Z^{-1}\Delta Z_{u}X\right|\left|F\right|+\left|\Delta Y_{u}-Z^{-1}\Delta Z_{u}Y\right|\left|E\right|\\ \;+\left|Z^{-1}\Delta Z_{l}\alpha \mu D^{\mu +1}e(t)\right|+\left|Z^{-1}\Delta Z_{l}\lambda e{\left(t\right)}^{2}sign(e(t))\right|) \end{array}$$ (21)

Obviously, by substituting Eq (21) into Eq (20), $\dot{L}<0$ will be observed. Actually, the reaching control achieves a stable sliding mode control system. The *u*_*s*_*(t)* can be defined as:

$$u_{s}(t)=K_{s}sign(s(t))$$ (22)

Where *K*_*s*_ *= diag* [*K*_*s1*_, *K*_*s2*_,*……*.., *K*_*sn*_] show reaching control gains.

## Novel compound fractional PID sliding mode control

The idea of FPID controller was proposed by Podlubny [32]. The FPID controller is much better than conventional PID controller. It has two additional control parameters defined as integration and differentiation orders which may enable the controller to provide the more flexibility and stability, which can be defined as:

$$u_{FPID}(t)=K_{p}e(t)+K_{i}D^{-\mu }e(t)+K_{d}D^{\mu }e(t)$$ (23)

**Fig 2:**
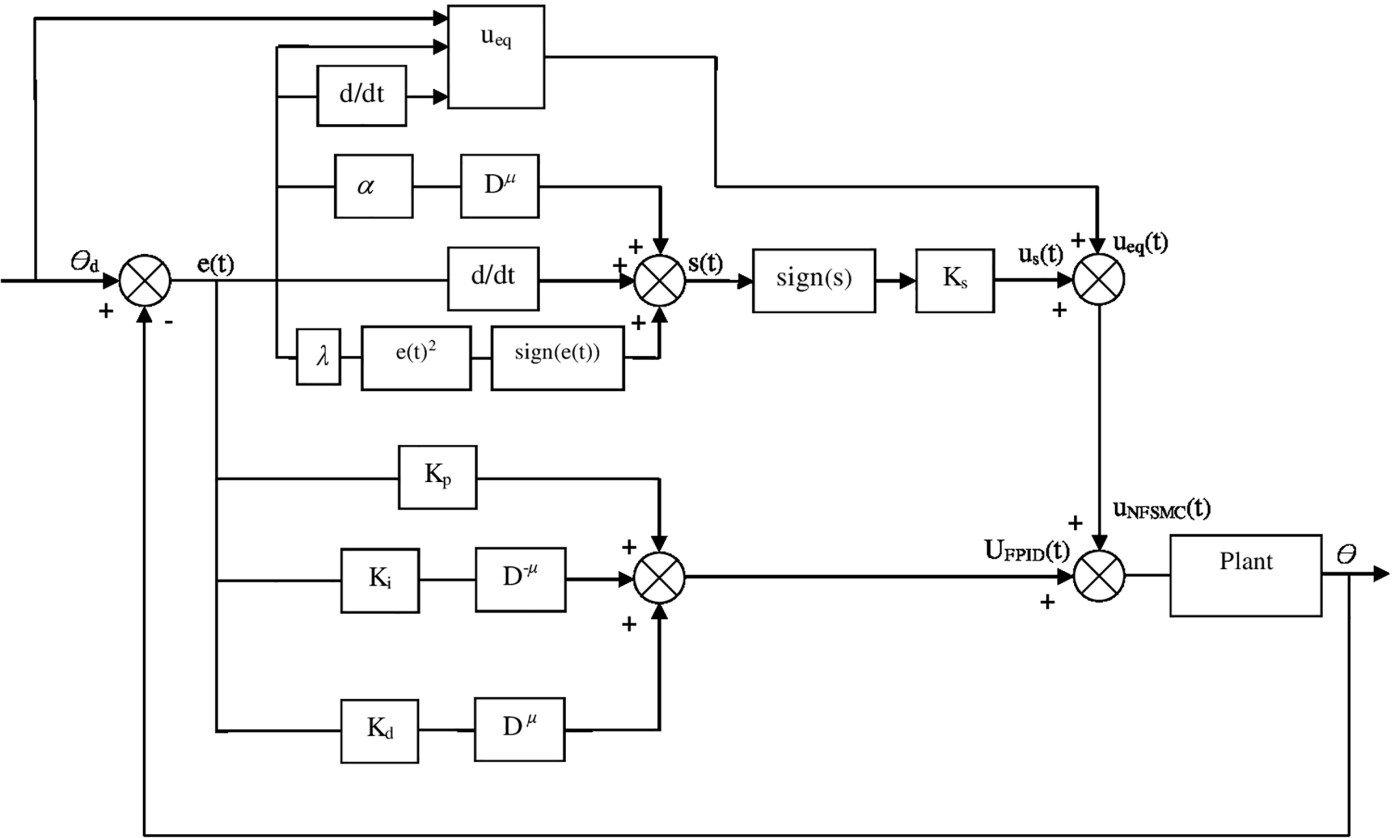
Block diagram of novel compound control system.

Where *K*_*p*_, *K*_*i*_, and *K*_*d*_ are proportional, integral, and derivative gains, respectively, and *μ* is fractional order operator. FPID and NFSMC are such a convenient controllers, which FPID has high tracking performance, and NFSMC is robust against external disturbances. Therefore, by combinig FPID and NFSMC, a novel control method will be created which benefits advantages of both controllers. The block diagram of new control method is illustrated in Fig 2, which can be defined as follows:

$$u(t)=u_{FPID}(t)+u_{NFSMC}(t)$$ (24)

By substituting Eq (24) and Eq (13) into Eq (16), it can shown as:

$$\begin{array}{l} \dot{L}(t)=s^{T}({\ddot{\theta }}_{d}+(V+\Delta V)\dot{\theta }+(W+\Delta W)G+(X+\Delta X)F\\ \;+(Y+\Delta Y)E-(Z+\Delta Z)u_{eq}(t)-(Z+\Delta Z)u_{s}(t)\\ \;-(Z+\Delta Z)u_{FPID}(t)+\alpha \mu D^{\mu +1}e(t)+\lambda e(t{)}^{2}sign(e(t))) \end{array}$$ (25)

According to Eq (23), Eq (25) can be denoted as:

$$\begin{array}{l} \dot{L}(t)=s^{T}({\ddot{\theta }}_{d}+(V+\Delta V)\dot{\theta }+(W+\Delta W)G+(X+\Delta X)F+(Y+\Delta Y)E\\ \;-(Z+\Delta Z)u_{eq}(t)-(Z+\Delta Z)u_{s}(t)-(Z+\Delta Z)K_{p}e(t)-(Z+\Delta Z)K_{i}D^{-\mu }e(t)\\ \;-(Z+\Delta Z)K_{d}D^{\mu }e(t)+\alpha \mu D^{\mu +1}e(t)+\lambda e(t{)}^{2}sign(e(t))) \end{array}$$ (26)

By substituting Eq (10) into Eq (26), it can be written as follows:

$$\begin{array}{l} \dot{L}(t)=s^{T}({\ddot{\theta }}_{d}+(V+\Delta V)\dot{\theta }+(W+\Delta W)G+(X+\Delta X)F+(Y+\Delta Y)E\\ \;-(Z+\Delta Z)(Z^{-1}({\ddot{\theta }}_{d}+V\dot{\theta }+WG+XF+YE+\alpha \mu D^{\mu +1}e\\ \;+\lambda e(t{)}^{2}sign(e(t))))-(Z+\Delta Z)u_{s}(t)-(Z+\Delta Z)K_{p}e(t)\\ \;-(Z+\Delta Z)K_{i}D^{-\mu }e(t)-(Z+\Delta Z)K_{d}D^{\mu }e(t)+\alpha \mu D^{\mu +1}e(t)\\ \;+\lambda e(t{)}^{2}sign(e(t))) \end{array}$$ (27)

Eq (27) can be demonstrated as:

$$\begin{array}{l} \dot{L}(t)=s^{T}({\ddot{\theta }}_{d}+V\dot{\theta }+\Delta V\dot{\theta }+WG+\Delta WG+XF+\Delta XF+YE+\Delta YE-{\ddot{\theta }}_{d}-V\dot{\theta }\\ \;-WG-XF-YE-\alpha \mu D^{\mu +1}e(t)-\lambda e(t{)}^{2}sign(e(t))-Z^{-1}\Delta Z{\ddot{\theta }}_{d}\\ \;-Z^{-1}\Delta ZV\dot{\theta }-Z^{-1}\Delta ZWG-Z^{-1}\Delta ZXF-Z^{-1}\Delta ZYE\\ \;-Z^{-1}\Delta Z\alpha \mu D^{\mu +1}e(t)-Z^{-1}\Delta Z\lambda e(t{)}^{2}sign(e(t))\\ \;-(Z+\Delta Z)u_{s}(t)+\alpha \mu D^{\mu +1}e(t)+\lambda e(t{)}^{2}sign(e(t))\\ \;-(Z+\Delta Z)K_{p}e(t)-(Z+\Delta Z)K_{i}D^{-\mu }e(t)-(Z+\Delta Z)K_{d}D^{\mu }e(t)) \end{array}$$ (28)

Simplyfing Eq (28) results in

$$\begin{array}{l} \dot{L}(t)=s^{T}(\Delta V\dot{\theta }+\Delta WG+\Delta XF+\Delta YE-Z^{-1}\Delta Z{\ddot{\theta }}_{d}-Z^{-1}\Delta ZV\dot{\theta }\\ \;-Z^{-1}\Delta ZWG-Z^{-1}\Delta ZXF-Z^{-1}\Delta ZYE-Z^{-1}\Delta Z\alpha \mu D^{\mu +1}e(t)\\ \;-Z^{-1}\Delta Z\lambda e(t{)}^{2}sign(e(t))-(Z+\Delta Z)u_{s}(t)-(Z+\Delta Z)K_{p}e(t)\\ \;-(Z+\Delta Z)K_{i}D^{-\mu }e(t)-(Z+\Delta Z)K_{d}D^{\mu }e(t)) \end{array}$$ (29)

It is noticed that tracking error will tend to zero(*e*(*t*)→0) when time goes to infinity (*t*→∞). Therefore, Eq (29) can be written as follows:

$$\begin{array}{l} \dot{L}(t)=s^{T}(\Delta V\dot{\theta }+\Delta WG+\Delta XF+\Delta YE-Z^{-1}\Delta Z{\ddot{\theta }}_{d}\\ \;-Z^{-1}\Delta ZV\dot{\theta }-Z^{-1}\Delta ZWG-Z^{-1}\Delta ZXF-Z^{-1}\Delta ZYE\\ \;-Z^{-1}\Delta Z\alpha \mu D^{\mu +1}e(t)-Z^{-1}\Delta Z\lambda e(t{)}^{2}sign(e(t))\\ \;-(Z+\Delta Z)u_{s}(t))\\ \;\leq s^{T}(-\left|Z^{-1}\Delta Z\right|\left|{\ddot{\theta }}_{d}\right|-\left|Z^{-1}\Delta ZV+\Delta V\right|\left|\dot{\theta }\right|+\left|\Delta W-Z^{-1}\Delta ZW\right|\left|G\right|\\ \;+\left|\Delta X-Z^{-1}\Delta ZX\right|\left|F\right|+\left|\Delta Y-Z^{-1}\Delta ZY\right|\left|E\right|-\left|Z^{-1}\Delta Z\alpha \mu D^{\mu +1}e(t)\right|\\ \;-\left|Z^{-1}\Delta Z\lambda e{\left(t\right)}^{2}sign(e(t))\right|)-s^{T}((Z+\Delta Z)u_{s}(t)) \end{array}$$ (30)

To ensure Eq (30) is less than zero, the reaching control law should be chosen as follows:

$$\begin{array}{l} u_{s}(t)=sign(s)(Z+\Delta Z{)}^{-1}(\left|Z^{-1}\Delta Z_{l}\right|\left|{\ddot{\theta }}_{d}\right|+\left|Z^{-1}\Delta Z_{l}V+\Delta V_{l}\right|\left|\dot{\theta }\right|\\ \;+\left|\Delta W_{u}-Z^{-1}\Delta Z_{u}W\right|\left|G\right|+\left|\Delta X_{u}-Z^{-1}\Delta Z_{u}X\right|\left|F\right|\\ \;+\left|\Delta Y_{u}-Z^{-1}\Delta Z_{u}Y\right|\left|E\right|+\left|Z^{-1}\Delta Z_{l}\alpha \mu D^{\mu +1}e(t)\right|\\ \;+\left|Z^{-1}\Delta Z_{l}\lambda e{\left(t\right)}^{2}sign(e(t))\right|) \end{array}$$ (31)

By substituting Eq (31) into Eq (30), It can be shown as:

$$\begin{array}{l} \dot{L}(t)\leq s^{T}(-\left|Z^{-1}\Delta Z\right|\left|{\ddot{\theta }}_{d}\right|-\left|Z^{-1}\Delta ZV+\Delta V\right|\left|\dot{\theta }\right|+\left|\Delta W-Z^{-1}\Delta ZW\right|\left|G\right|\\ \;+\left|\Delta X-Z^{-1}\Delta ZX\right|\left|F\right|+\left|\Delta Y-Z^{-1}\Delta ZY\right|\left|E\right|-\left|Z^{-1}\Delta Z\alpha \mu D^{\mu +1}e(t)\right|\\ \;-\left|Z^{-1}\Delta Z\lambda e{\left(t\right)}^{2}sign(e(t))\right|-sign(s)\left|Z^{-1}\Delta Z_{l}\right|\left|{\ddot{\theta }}_{d}\right|\\ \;-sign(s)\left|Z^{-1}\Delta Z_{l}V+\Delta V_{l}\right|\left|\dot{\theta }\right|-sign(s)\left|\Delta W_{u}-Z^{-1}\Delta Z_{u}W\right|\left|G\right|\\ \;-sign(s)\left|\Delta X_{u}-Z^{-1}\Delta Z_{u}X\right|\left|F\right|-sign(s)\left|\Delta Y_{u}-Z^{-1}\Delta Z_{u}Y\right|\left|E\right|\\ \;-sign(s)\left|Z^{-1}\Delta Z_{l}\alpha \mu D^{\mu +1}e(t)\right|-sign(s)\left|Z^{-1}\Delta Z_{l}\lambda e{\left(t\right)}^{2}\sin(e(t))\right|) \end{array}$$ (32)

The Eq (32) can be rewritten as follow:

$$\begin{array}{l} \dot{L}(t)\leq s^{T}((\underset{}{\underset{︸}{-\left|Z^{-1}\Delta Z\right|-sign(s)\left|Z^{-1}\Delta Z_{l}\right|)\left|{\ddot{\theta }}_{d}\right|}}+(-\left|Z^{-1}\Delta ZV+\Delta V\right|\\ \;-sign(s)\left|Z^{-1}\Delta Z_{l}V+\Delta V_{l}\right|)\left|\dot{\theta }\right|+(\left|\Delta W-Z^{-1}\Delta ZW\right|\\ \;-sign(s)\left|\Delta W_{u}-Z^{-1}\Delta Z_{u}W\right|)\left|G\right|+(\left|\Delta X-Z^{-1}\Delta ZX\right|\\ \;-sign(s)\left|\Delta X_{u}-Z^{-1}\Delta Z_{u}X\right|)\left|F\right|+(\left|\Delta Y-Z^{-1}\Delta ZY\right|\\ \;-sign(s)\left|\Delta Y_{u}-Z^{-1}\Delta Z_{u}Y\right|)\left|E\right|+(-\left|Z^{-1}\Delta Z\alpha \mu D^{\mu -1}e(t)\right|\\ \;-sign(s)\left|Z^{-1}\Delta Z_{l}\alpha \mu D^{\mu +1}e(t)\right|)+(-\left|Z^{-1}\Delta Z\lambda e{\left(t\right)}^{2}\sin(e(t))\right|\\ \;-sign(s)\left|Z^{-1}\Delta Z_{l}\lambda e{\left(t\right)}^{2}\sin(e(t))\right|)\\ \; \end{array}$$ (33)

According to Eq (12), *s*(*t*) ≠ 0. Therefore, *sign(s)* is equal to 1 or -1. We will consider one term of Eq (33), which all other terms will be proved by this process. In Eq (33), $(-\left|Z^{-1}\Delta Z\right|-sign(s)\left|Z^{-1}\Delta Z_{l}\right|)\left|{\ddot{\theta }}_{d}\right|$ Can be explained as:

It can be taken into considerations that |Δ*Z*_*l*_|<|Δ*Z*|<|Δ*Z*_*u*_|. As a result of this, |*Z*^−1^Δ*Z*|>|*Z*^−1^Δ*Z*_*l*_|. Consequently, (−|*Z*^−1^Δ*Z*|−*sign*(*s*)|*Z*^−1^Δ*Z*_*l*_|)<0. All other term can be proved by that process which are less than zero. Therefore, $\dot{L}<0$ will be observed. Actually, the reaching control achieves a stable sliding mode control system. The us(t) can be defined as:

$$u_{s}(t)=K_{s}sign(s(t))$$ (34)

Where *K*_*s*_ *= diag* [*K*_*s1*_, *K*_*s2*_,*……*.., *K*_*sn*_] show reaching control gains.

## Implementation of fractional order operator

Fractional differential equations and fractional order derivatives have been widely used in recent years [33]. The fractional order derivatives become popular because it can be used in modeling techniques, and computational methods for the numerical solution of these models. Consequently, due to its abilities, it can be used appropriately in control methods. The Geunwald- Letnikov operator is one type of fractional operators, which can be defined as [34]:

$${}_{a}D{}_{t}^{\mu }\underset{h\to 0}{\lim}\frac{1}{h^{\mu }}\sum_{r=0}^{\left[\frac{t-a}{h}\right]}{\left(-1\right)}^{r}\left(\begin{array}{c} n\\ r \end{array}\right)f(t-rh)$$ (35)

Where *a* and *t* are the limits of operator and [*t-a/h*] is the integer part. n is the integer value which satisfies the condition *n-1*<*μ*<*n*.

The value of the binomial coefficient is shown by

$$\left(\begin{array}{c} n\\ r \end{array}\right)=\frac{\Gamma (n+1)}{\Gamma (r+1)\Gamma (n-r+1)}$$ (36)

The Gamma function utilized in Eq (36) can be defined as follows:

$$\Gamma (x)=\int_{0}^{\infty }t^{x-1}e^{-t}dt,\;R(z)>0$$ (37)

This definition is significantly appropriate in obtaining a numerical solution of fractional differential equations.

## Simulation results

**Fig 3:**
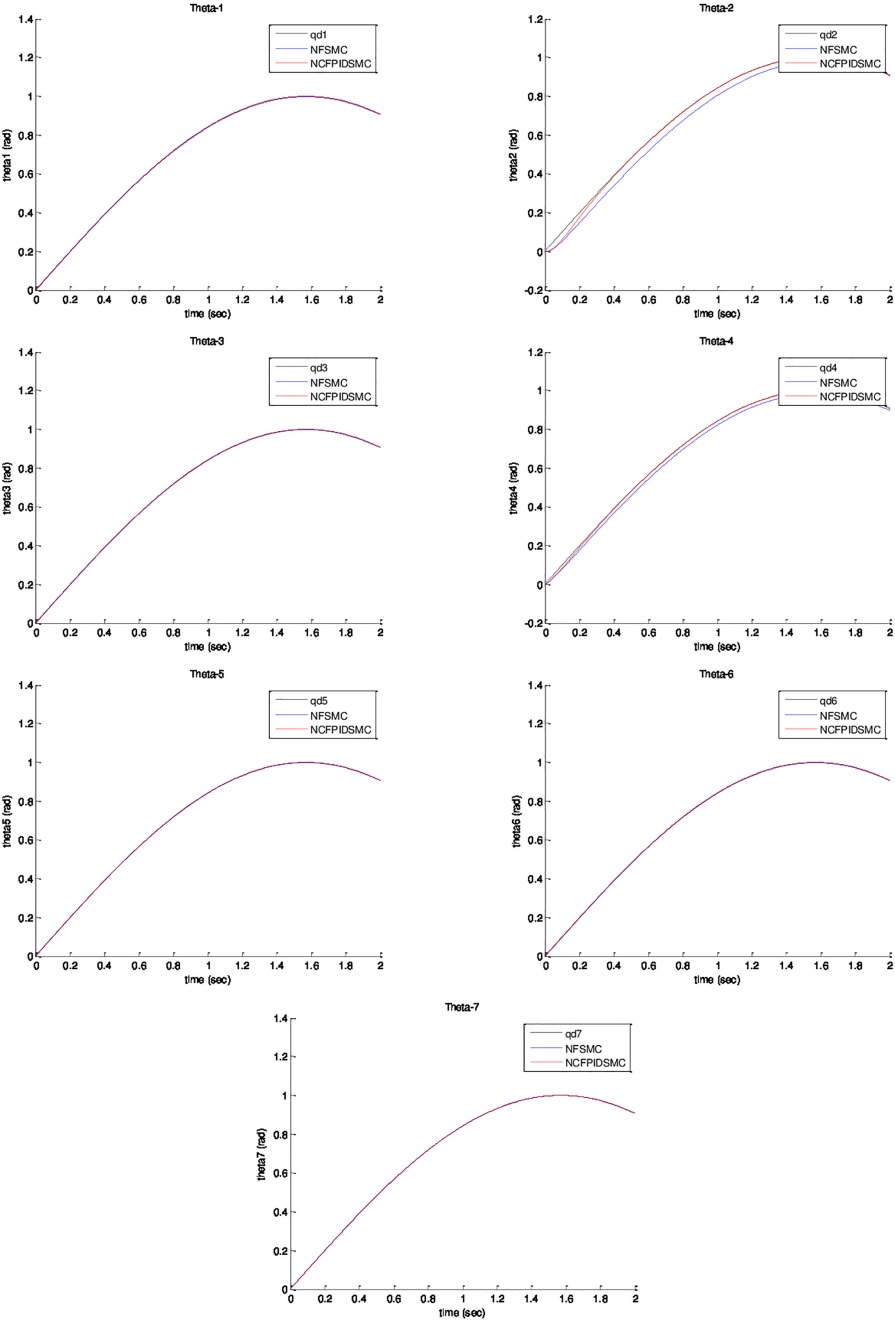
Position tracking of joints under NFSMC and NCFPIDSMC.

**Fig 4:**
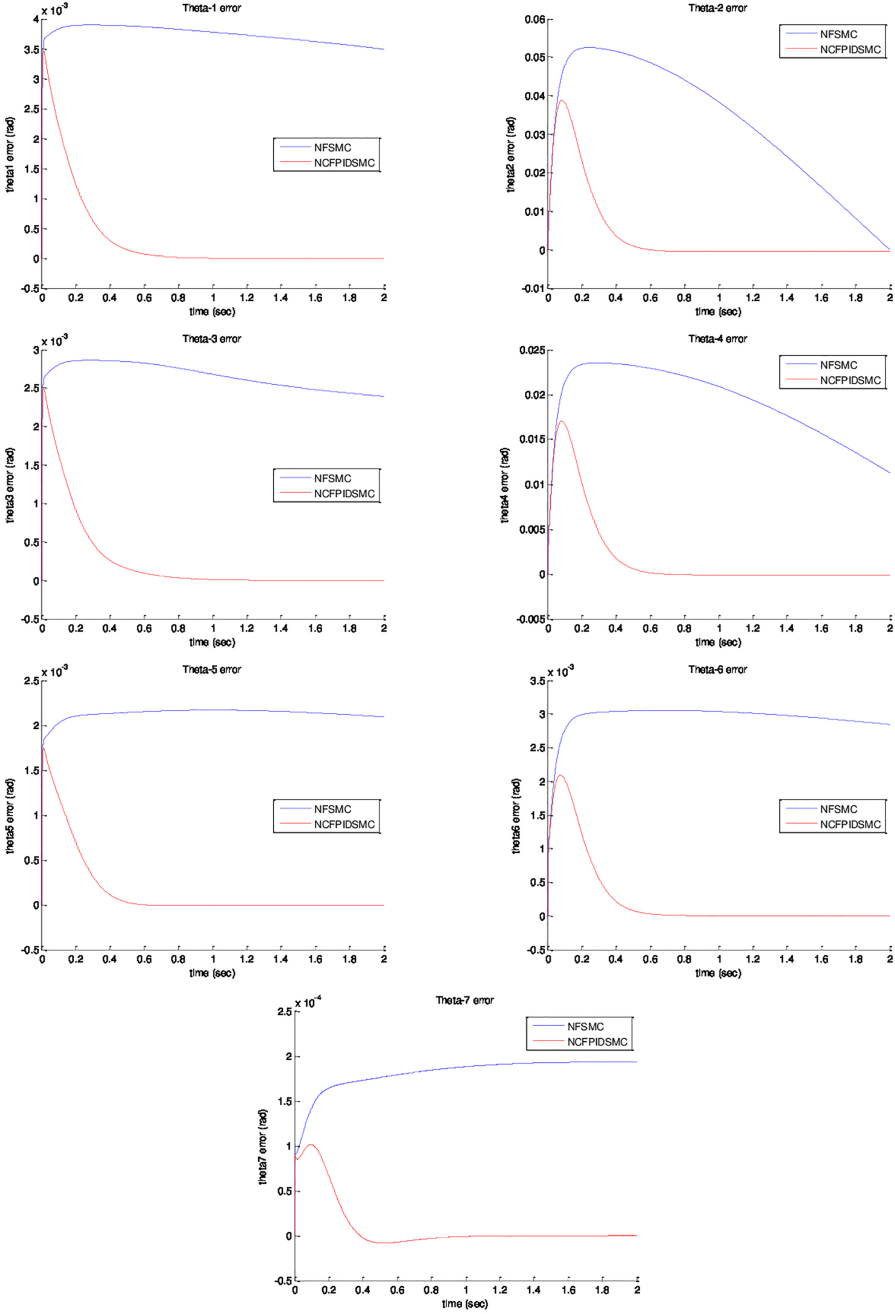
Position tracking error of joints under NFSMC and NCFPIDSMC.

**Fig 5:**
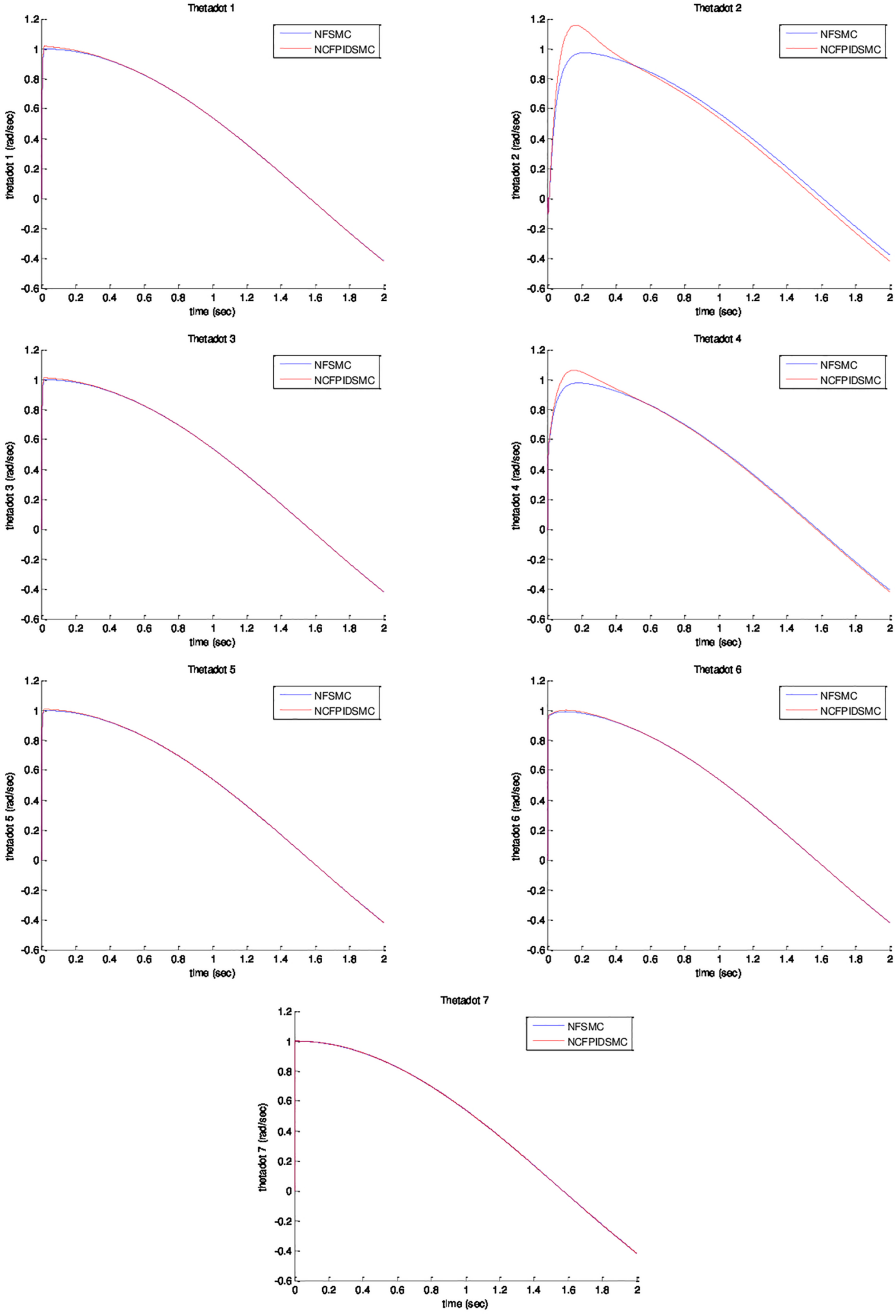
Velocity of joints under NFSMC and NCFPIDSMC.

**Fig 6:**
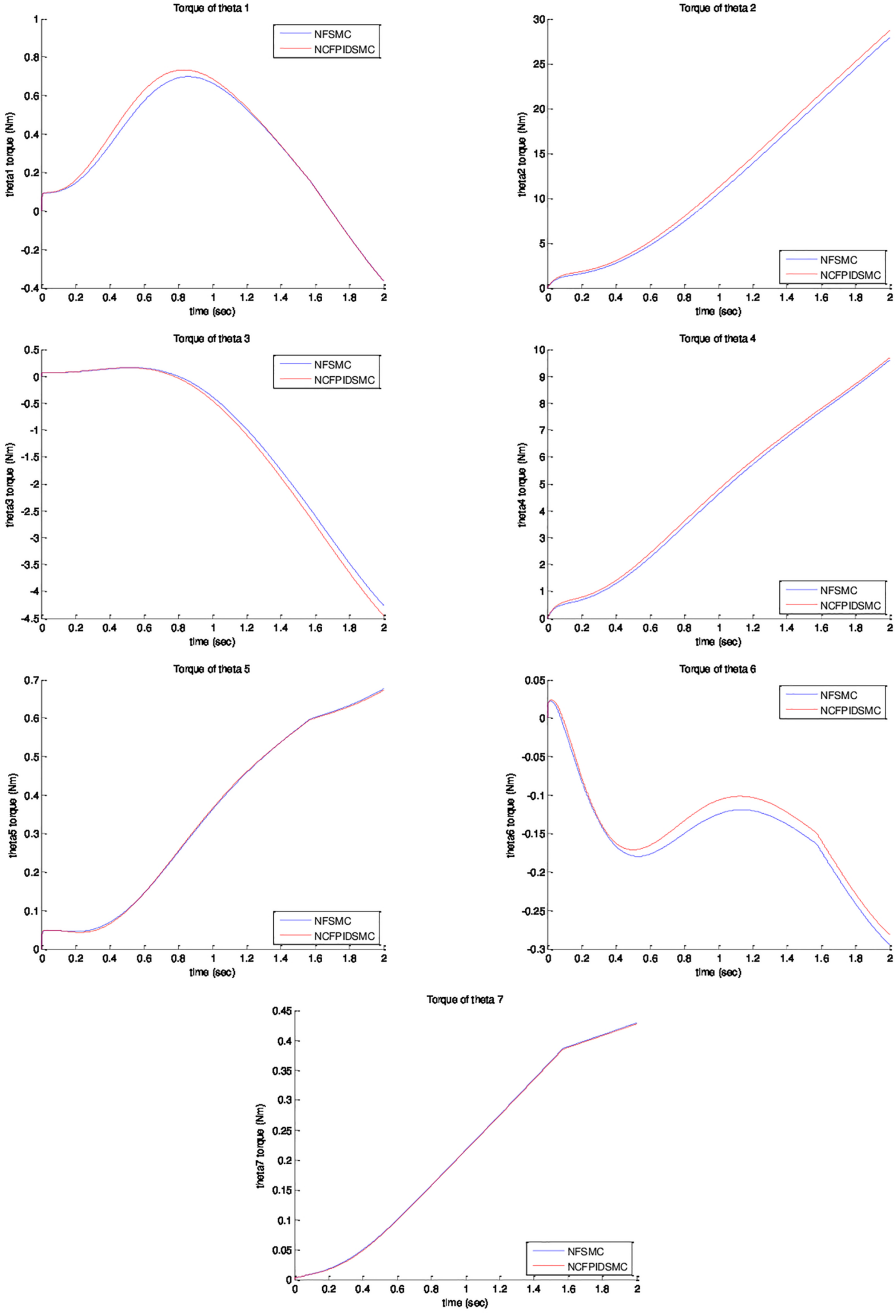
Control effort using NFSMC and NCFPIDSMC.

The numerical simulations have been done on ETS-MARSE robot model. The FPID controller parameters are chosen as *Kp = diag(150*, *150*, *150*, *150*, *150*, *150*, *150)*, *K*_*i*_ *= diag(30*, *30*, *30*, *30*, *30*, *30*, *30)*, *Kd = diag(80*, *80*, *80*, *80*, *80*, *80*, *80)*, and *μ = 1*.*5*. The new fractional sliding mode surface parameters are selected as *α = 30*, *λ = 15*, and *K*_*s*_ *= diag(25*, *25*, *25*, *25*, *25*, *25*, *25)*. The desired trajectory tracking is determined by *θ*_*d1*_ *= θ*_*d2*_ *= θ*_*d3*_ *= θ*_*d4*_ *= θ*_*d5*_ *= θ*_*d6*_ *= θ*_*d7*_ *= sin(t)*. The initial value of system are chosen as *θ*_*1*_*(0) = θ*_*2*_*(0) = θ*_*3*_*(0) = θ*_*4*_*(0) = θ*_*5*_*(0) = θ*_*6*_*(0) = θ*_*7*_*(0) = 0*, and ${\dot{\theta }}_{1}(0)={\dot{\theta }}_{2}(0)={\dot{\theta }}_{3}(0)={\dot{\theta }}_{4}(0)={\dot{\theta }}_{5}(0)={\dot{\theta }}_{6}(0)={\dot{\theta }}_{7}(0)=0$. Physical parameters of ETS-MARSE robot are tabulated in Table 3. Fig 3 shows trajectory tracking of joints under NFSMC and NCFPIDSMC. According to that figure, NCFPIDSMC has better performance in tracking in comparison with NFSMC. Therefore, the proposed control tracks the desired trajectory conveniently. Fig 4 illustrates tracking error of joints under FSMC and NCFPIDSMC. The proposed control method tracking error converges to zero in limited amount of time in comparison with NFSMC. It can be noticed that maximum overshoot in NCFPIDSMC is closed to zero. Also, the proposed control method is better than NFSMC in terms of settling time and convergence time, which enhances the dynamic behavior of the ETS-MARSE robot and confirms that the proposed control method can guarantee the asymptotical stability of the control system. Fig 5 shows velocity of joints under NFSMC and NCFPIDSMC. Fig 6 illustrates control effort using NSFMC and NCFPIDSMC. It shows that the control input is bounded and convergent.

**Table 3:** Physical parameters of ETS- MARSE.

Joints	Mass (Kg)	Center of mass (m)	Link length (m)
1	3.475	0.0984	0.145
2	3.737	0.1959	0
3	0	0	0.25
4	2.066	0.163	0
5	0	0	0.267
6	0.779	0.121	0
7	0.496	0.0622	0

By observing all figures from simulation, the proposed control law has the best performance in comparison with NFSMC, and verified all assumption of designing that controller.

### Robustness testing: Random noise suppression

By designing an appropriate fractional sliding mode surface, the best performance will be obtained by that process. The main advantages of the NFSMC is its robustness against external disturbances, but it doesn’t have suitable tracking performance. By combining FPID controller and NFSMC, a new hybrid control system will be obtained, which benefits both controller advantages. The FPID controller continuously calculates an error value e(t) and applied a correction based *K*_*p*_*e*(*t*),*K*_*i*_*D*^−*μ*^*e*(*t*) and *K*_*d*_*D*^*μ*^*e*(*t*) terms. This issue can improve tracking performance, reduce chattering phenomenon, maximum overshoot.

The ETS-MARSE robot has been constantly encountered with external disturbances and model uncertainties, which are included as different hieghts, masses, and disease conditions such as different degrees of spasticity. Therefore, a random noise is applied to verify robustness of the proposed control method against external disturbances and model uncertainties. A random noise with standard deviation of 0.05 is applied on ETS-MARSE as follows:

E(t)=0.5*randn(1,1) (38)

**Fig 7:**
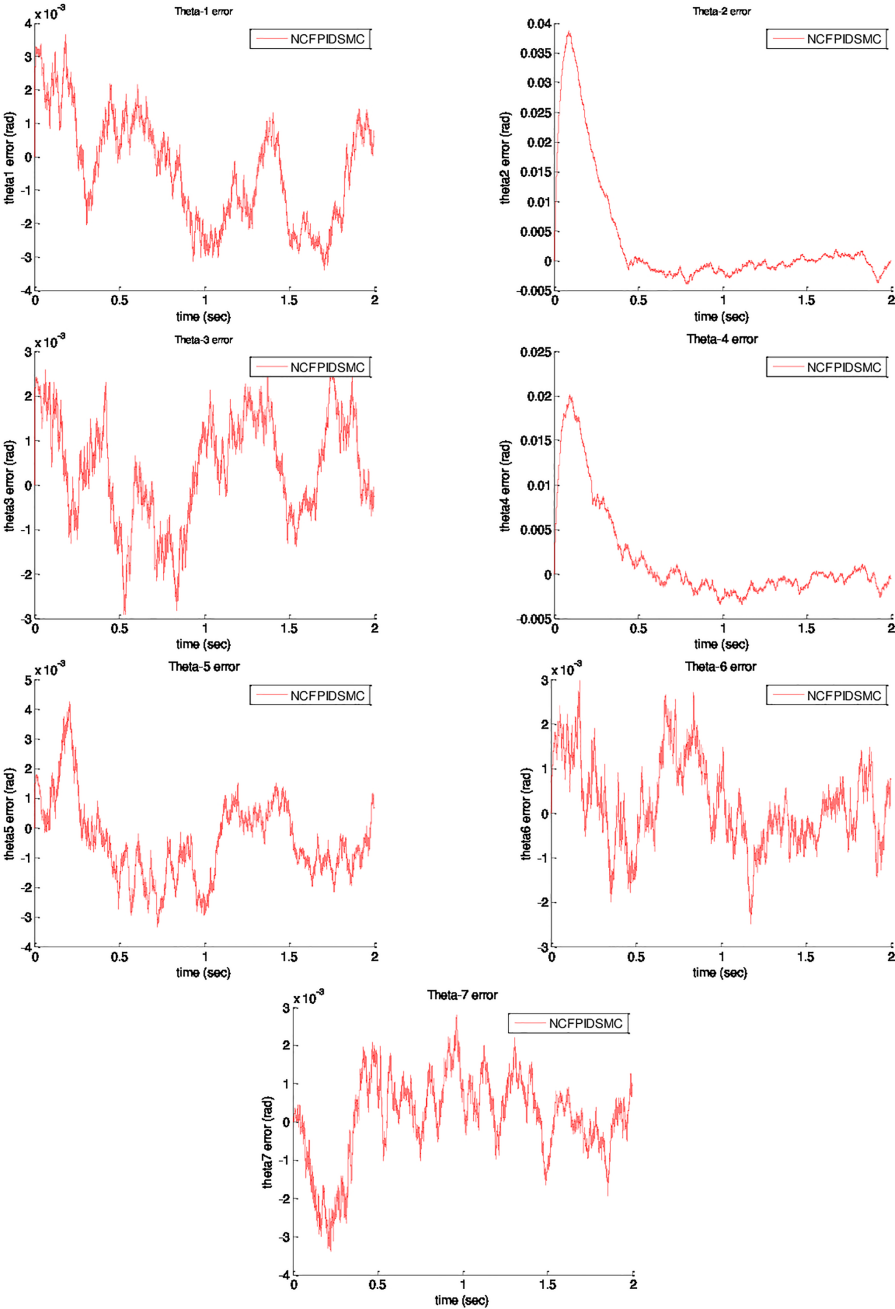
Robustness verification of NCFPIDSMC under random noise application.

Fig 7 shows that NCFPIDSMC is completely able to suppress the noise. It can be taken into considerations that the proposed control method will not be destabilized when encountered with random noise application. It shows that the proposed control method includes both robustness and high tracking performance.

## Conclusion

This paper proposed a novel robust control method for control of a 7-DOF exoskeleton robot named ETS-MARSE. First, a NFSMC controller proposed which is robust against external disturbances and model uncertainties, but low tracking performance is its main drawback. FPID controller is not robust against external disturbances, but it has high tracking performance. Next, a new compound control method designed which has the advantages of NFSMC and FPID controllers. Simulation results verified the effectiveness of proposed control method in terms of low maximum overshoot, low settling time, and faster convergence time. Finally, the robustness of the proposed control method verified by a random noise.
